# A case report describing the use of systemic bevacizumab in the treatment of recurrent respiratory papillomatosis with pulmonary involvement

**DOI:** 10.1002/rcr2.1246

**Published:** 2023-11-22

**Authors:** Amy O'Brien, Eskandarain Shafuddin

**Affiliations:** ^1^ Department of Respiratory Medicine Te Whatu Ora Waikato Hospital Hamilton New Zealand

**Keywords:** avastin, bevacizumab, human viruses, papilloma, papilloma, recurrent respiratory papillomatosis

## Abstract

Recurrent respiratory papillomatosis (RRP) is a rare disease characterized by recurrent papilloma along the aerodigestive tract. In this case, we describe a 16‐year‐old with longstanding laryngeal RRP secondary to vertical transmission of human papillomavirus (HPV) who presented with symptomatic pulmonary involvement and was successfully treated with systemic bevacizumab. The case describes the clinical and radiological improvement with therapy as well as the adverse effects that occurred and resolved with dose adjustments.

## Graphical abstract

Recurrent respiratory papillomatosis with is a rare disease caused by HPV 6 and 11. This article describes successful treatment of recurrent respiratory papillomatosis with pulmonary involvement with systemic bevacizumab.

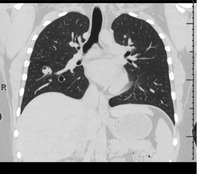

## INTRODUCTION

Recurrent respiratory papillomatosis (RRP) is a rare disease commonly caused by human papillomavirus (HPV) 6 and 11. It is characterized by recurrent exophytic papilloma primarily in the larynx, and occasionally in other parts of the aerodigestive tract. It has an estimated incidence of 1.8 per 100,000 adults and 4.3 per 100,000 children.^1^ Lung involvement is rare and seen in approximately 1% of cases.^2^ Currently, no cure exists for RRP and the standard treatment goals are to limit the papilloma burden by surgical excision or laser ablation.

## CASE REPORT

A 16‐year‐old Māori female with known RRP since infancy, was referred to the respiratory clinic after a chest x‐ray was performed in the emergency department (ED) revealing an increasing right mid‐zone lesion.

The patient was diagnosed with RRP at 11 months old after presenting with a hoarse voice. A laryngoscopy at the time showed laryngeal papilloma. The histopathology of this showed benign squamous cell tissue which was positive for human papilloma virus 11. The patient's mother had a history of genital warts, including cervical warts, at the time of the patient's birth.

Over the next 16 years, 115 laryngoscopies were performed for micro‐debridement with laser debulking of recurrent papillomas. Her main symptoms included dysphonia and cough. When the patient was 8 years old, a computed tomography (CT) scan of the chest revealed two thin‐walled cysts in the right upper and lower lobes. No respiratory symptoms were reported at this time and baseline spirometry was normal.

The patient did not have any other medical history. Her regular medications at the time of review included combined oral contraceptives and melatonin. She had trialled intra‐lesional Cidofovir and intra‐lesional Bevacizumab in the past without success.

At age 16 the patient presented to our ED with cough and haemoptysis. A chest x‐ray showed an enlarging right mid‐zone nodule., The patient did not have any infective symptoms and her examination and blood workup were unremarkable. This was her fourth presentation in 12 months to ED with a cough and sore throat. She was referred to the adult respiratory service for further workup. In the 2 years prior to the first adult respiratory review, the patient underwent nine laryngoscopies for micro‐debridement.

**FIGURE 1:**
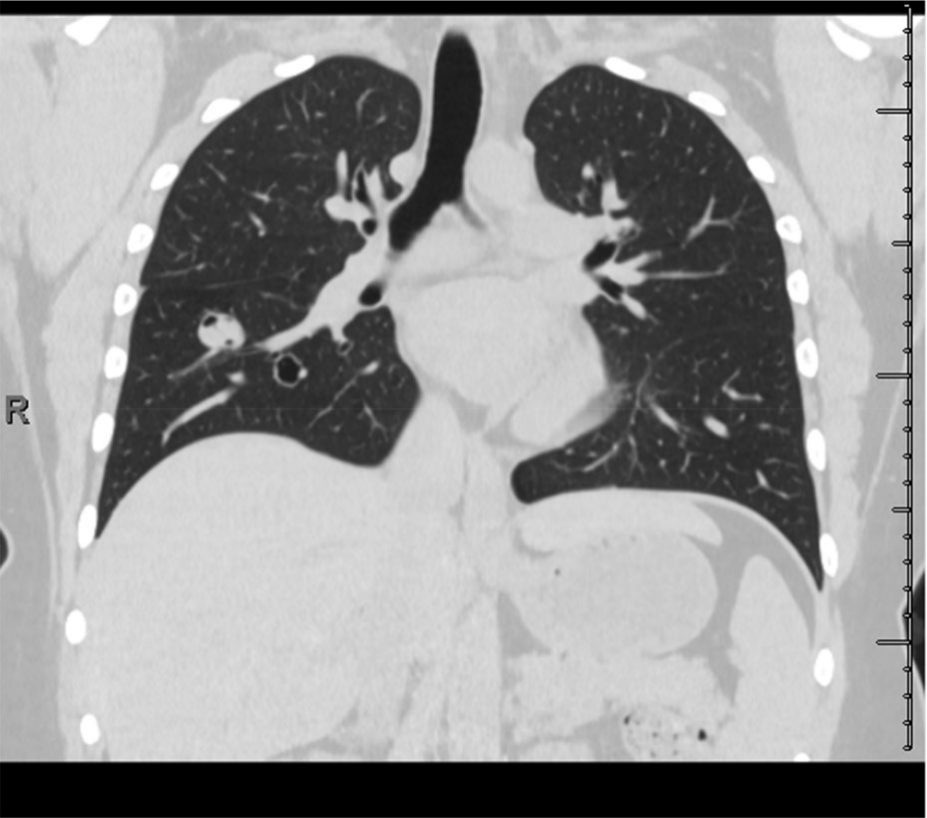
The figure shows a computed tomography (CT) scan of the patient's chest prior to starting treatment with bevacizumab. The CT chest shows a new 14 mm lobulated pulmonary nodule abutting the known right lower lobe cyst, a new multi‐cystic lesion in the right lower lobe.

A CT chest showed a new 14 mm lobulated pulmonary nodule abutting the known right lower lobe cyst, a new multi‐cystic lesion in the right lower lobe and a stable thin‐walled cyst in the right upper lobe (Figure 1). These were non avid on a Positron emission tomography (PET)‐CT scan. A bronchoscopy was performed, histopathology of the washings and transbronchial biopsy demonstrated benign epithelial cells and the microscopy was negative for infection.

Following these investigations, a multidisciplinary discussion was held regarding the ongoing management of this case. At this time, case reports were emerging with promising results of systemic bevacizumab for RRP.^3^ Funding from the New Zealand Pharmaceutical Management Agency (Pharmac) was approved for systemic bevacizumab treatment.

The patient agreed to a trial of bevacizumab 7 mg/kg, initially on a three‐weekly basis with the intervals between doses to be increased based on advice from oncologists and expert opinion in Australia and the United States. At the time of starting treatment, the patient weighed 96 kg and received 700 mg of Bevacizumab intravenously.

Following her third infusion, the patient presented to ED with nausea, vomiting and diarrhoea. She required an admission for intravenous fluids. The dose of bevacizumab was reduced to 5 mg/kg (500 mg) to reduce the side effect profile. It was noted at this time the patient's cough had resolved. The patient then completed three further doses of 5 mg/kg bevacizumab at six weekly intervals with complete resolution of RRP‐related symptoms. After the sixth infusion, the infusion was extended to 9 weeks for three doses. Following the ninth infusion, IV Bevacizumab will be given 12‐weekly for three doses with the aim of maintaining the patient on infusions 16‐weekly.

**FIGURE 2:**
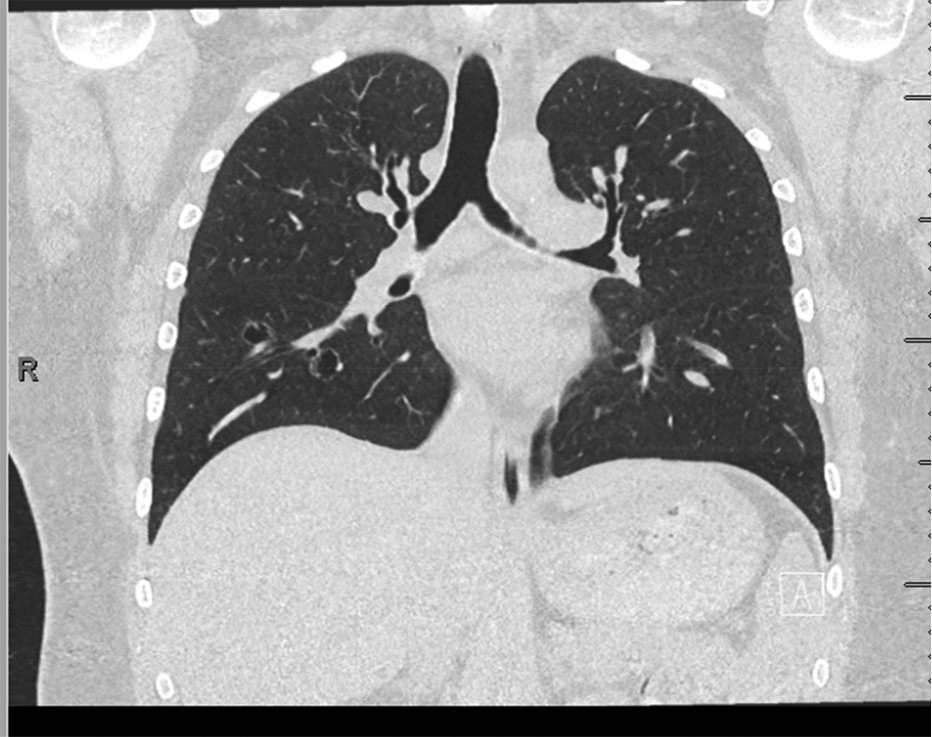
The figure shows a computed tomography scan of the patient's following 9 weeks of IV bevacizumab. The 14 mm right lower lobe nodule had resolved.

The patient was reviewed 7 months into bevacizumab treatment in the respiratory clinic and reported the cough and haemoptysis had resolved. During the first 6 months of treatment, the patient had a mild influenza A infection which was not related to Bevacizumab treatment and she did not require oxygen or anti‐viral therapy. The patient did not have any further ED presentations with cough or require further antibiotics for respiratory tract infections. A CT Chest had been performed 9 weeks after initiating treatment which showed a reduction in the size of the two cystic lesions and complete resolution of the 14 mm lobulated lesion in the right lower lobe (Figure 2).

The patient has had symptomatic improvement from a laryngeal perspective and a direct laryngoscopy performed by the otolaryngologist 7 months into treatment showed complete remission of disease without any remaining papilloma. The patient has not required micro‐debridement or laser debulking of papillomas since starting bevacizumab treatment. The patient is being monitored six‐monthly by the respiratory and otaralyngology clinics.

## DISCUSSION

RRP is a challenging disease that is associated with significant morbidity such as voice disturbance and airway obstruction. The current mainstay of treatment is surgery with cold instruments, laser, or micro‐debridement to remove papillomas and to prevent disease spread. On average, patients with RRP will require four surgeries per year to achieve control. Up to 20% of patients require adjuvant systemic treatment due to inadequate disease control by surgery alone.^4^

Bevacizumab is a humanized monoclonal antibody against vascular endothelial growth factor A (VEGF‐A). Systemic bevacizumab has been shown to reduce surgical frequency by indirectly inhibiting papilloma growth. The papilloma epithelium and underlying vascular epithelium show strong expression of VEGF, Vascular endothelial growth factor receptor‐1 (VEGFR‐1) and VEGFR‐2.^5^

A systematic review by Pogoda et al of all case series to date of systemic bevacizumab in the treatment of RRP found that 95% of the 43 patients treated with systemic bevacizumab responded to treatment with a reduction in surgery, 56% of all patients in this series no longer required surgery.^3^ The treatment was well tolerated, and the mean follow‐up was 21.6 months. Two recent case series have described promising long‐term response and tolerance in 35 patients with pulmonary RRP treated with systemic bevacizumab.^6^, ^7^ Both case series showed that most patients had a sustained response to treatment with a reduction in patient reported symptoms and need for endoscopic procedures. The most common side effects in both series were hypertension, headache, nausea, and vomiting. The mechanism for the adverse events is not fully understood but hypertension may be dose dependant when patients are started on higher doses (15 mg/kg per infusion). In our case, the patient initially suffered from gastrointestinal upset but following a dose reduction, the patient has tolerated the infusions without any significant side effects. The dosing was calculated based on expert opinions from an international colleague at the John Hopkins Hospital as well as advice from an oncologist at Auckland City Hospital with previous experience of Bevacizumab in upper airway RRP. The initial dosing in our case was lower than the regimens in the case series where the patients were started on 10–15 mg/kg Bevacizumab. Our patient has not required endoscopic interventions in the 7 months following initiation of systemic Bevacizumab and has shown clinical and radiological improvement in RRP. To date, she has not experienced other common side effects such as hypertension and headaches.

This case adds to the growing literature on the use of systemic treatments for RRP. To our knowledge, this is the first reported case of bevacizumab treatment for lung involvement of RRP in New Zealand or Australia.

## AUTHOR CONTRIBUTIONS

Dr Amy O'Brien, Respiratory Registrar, Te Whatu Ora Waikato Hospital, New Zealand. First author. Dr Eskandarain Shafuddin, Consultant Respiratory Physician, Te Whatu Ora Waikato Hospital, New Zealand. Supervisor.

## CONFLICT OF INTEREST STATEMENT

None declared.

## ETHICS STATEMENT

The authors declare that appropriate written informed consent was obtained for the publication of this manuscript and accompanying images.

## Data Availability

Data sharing is not applicable to this article as no new data were created or analysed in this study.
